# Divergent role of nitric oxide in insulin-stimulated aortic vasorelaxation between low- and high-intrinsic aerobic capacity rats

**DOI:** 10.14814/phy2.12459

**Published:** 2015-07-21

**Authors:** Jacqueline M Crissey, Jaume Padilla, Victoria J Vieira-Potter, Pamela K Thorne, Lauren G Koch, Steven L Britton, John P Thyfault, M Harold Laughlin

**Affiliations:** 1 Nutritional Sciences, University of TexasAustin, Texas; 2 Nutrition and Exercise Physiology, University of MissouriColumbia, Missouri; 3 Dalton Cardiovascular Research Center, University of MissouriColumbia, Missouri; 4 Child Health, University of MissouriColumbia, Missouri; 5 Biomedical Sciences, University of MissouriColumbia, Missouri; 6 Department of Anesthesiology, University of MichiganAnn Arbor, Michigan; 7 Department of Molecular and Integrative Physiology, Kansas University Medical CenterKansas, Kansas; 8 Medical Pharmacology and Physiology, University of MissouriColumbia, Missouri

**Keywords:** Aerobic capacity, nitric oxide, vascular reactivity to insulin

## Abstract

Low-intrinsic aerobic capacity is associated with increased risk for cardiovascular and metabolic diseases and is a strong predictor of early mortality. The effects of intrinsic aerobic capacity on the vascular response to insulin are largely unknown. We tested the hypothesis that rats selectively bred for a low capacity to run (LCR) exhibit vascular dysfunction and impaired vascular reactivity to insulin compared to high capacity running (HCR) rats. Mature female LCR (*n* = 21) and HCR (*n* = 17) rats were maintained under sedentary conditions, and in vitro thoracic aortic vascular function was assessed. LCR exhibited greater body mass (13%), body fat (35%), and subcutaneous, perigonadal, and retroperitoneal adipose tissue mass, than HCR. During an intraperitoneal glucose tolerance test, glucose area under the curve (AUC) was not different but insulin AUC was 2-fold greater in LCR than HCR. Acetylcholine and insulin-stimulated aortic vasorelaxation was significantly greater in LCR (65.2 ± 3.8%, and 32.7 ± 4.1%) than HCR (55.0 ± 3.3%, and 16.7 ± 2.8%). Inhibition of nitric oxide synthase (NOS) with L-NAME entirely abolished insulin-mediated vasorelaxation in the aorta of LCR, with no effect in HCR. LCR rats exhibited greater expression of Insulin Receptor protein, lower Endothelin Receptor-A protein, a down-regulation of transcripts for markers of immune cell infiltration (CD11C, CD4, and F4/80) and up-regulation of pro-atherogenic inflammatory genes (VCAM-1 and MCP-1) in the aorta wall. Contrary to our hypothesis, low-aerobic capacity was associated with enhanced aortic endothelial function and NO-mediated reactivity to insulin, despite increased adiposity and evidence of whole body insulin resistance.

Aerobic capacity, independent of physical activity, is a strong predictor of cardiovascular disease and overall mortality (Blair et al. 1989a, 1989b; Chang and Froelicher 1994; Pate et al. 1995; Myers et al. 2002). Low-aerobic capacity per se is associated with greater risk of death than other more established risk factors including physical inactivity, hypertension, smoking, and type 2 diabetes. The inverse relationship between aerobic capacity (i.e., aerobic fitness) and cardiovascular disease and mortality is observed in both men and women, and is not confounded by age or other risk factors. (Powell et al. 1987; Haskell et al. 1992; Blair et al. 1995).

Insulin resistance is also a major independent cardiovascular disease risk factor and is associated with all-cause mortality (Ford 2005; Cersosimo and DeFronzo 2006; Reddy et al. 2010). Insulin resistance is characterized by a blunted ability of peripheral tissues to regulate glucose homeostasis in response to insulin. While endothelial dysfunction is considered a precursor of vascular disease, evidence now indicates that vascular insulin resistance is also a prominent component of hypertension, coronary artery disease, and atherosclerosis (Reaven 1993). In healthy persons, insulin has important cardiovascular actions, including increased blood flow and capillary recruitment in skeletal muscle (Muniyappa et al. 2007). Abnormalities in vascular reactivity to insulin are present in obese, physically inactive, and type 2 diabetic humans and animals. Impairments in vascular sensitivity to insulin with disease contribute to reduced skeletal muscle glucose uptake by limiting perfusion and thus delivery of glucose and insulin (Laakso et al. 1992; Wallis et al. 2002; Kim et al. 2006; Mikus et al. 2010, 2012; Martin et al. 2012). While increased physical activity and exercise can improve the vasomotor response to insulin in animals and humans displaying a disease phenotype (Mikus et al. 2010, 2012; Martin et al. 2012; Crissey et al. 2014), the role of intrinsic aerobic capacity, independent of regular exercise, is relatively unknown.

In the absence of exercise training, genetic inheritance putatively accounts for up to 60–70% of the variation in intrinsic aerobic capacity in humans (Bouchard et al. 1986). Data from a novel rodent model developed by Koch and Britton demonstrate that low-aerobic capacity can be inherited, and leads to impaired metabolic health and risk factors for cardiovascular disease, independent of chronic exercise effects (Hussain et al. 2001; Koch and Britton 2001; Wisloff et al. 2005; Lessard et al. 2009). In this model, rats were selectively bred for high- or low-treadmill endurance running (performance on three-graded treadmill exercise tests) over several generations, resulting in two strains of rats with ∼30% difference in aerobic capacities (Wisloff et al. 2005). The sedentary low-capacity runners (LCR) display cardiovascular risk factors consistent with the metabolic syndrome including gains in visceral adiposity, increased blood pressure, dyslipidemia, microvascular endothelial dysfunction, and insulin resistance (Wisloff et al. 2005; Noland et al. 2007; Thyfault et al. 2009; Naples et al. 2010). Similar to the higher life expectancy and reduced disease and mortality risk observed in humans with high-aerobic capacity (Kujala et al. 2001; Aarnio et al. 2002), LCR have a 45% shorter median lifespan than the high-capacity running (HCR) animals (Koch et al. 2011). Together, these data support the hypothesis that low-aerobic capacity can underlie disease risks and reduce longevity. Since, these contrasting aerobic capacities occur in a sedentary, cage-activity-only condition (Hoydal et al. 2007) an advantage of this model is that it is free from the confounding influence of exercise training on aerobic capacity and disease risk (Wisloff et al. 2005; Bernal-Mizrachi and Semenkovich, 2006). This polygenic model of disease more accurately mimics the pathology of human chronic metabolic disease(s) than single gene mutations, making it an ideal model to study the interaction between aerobic capacity, and chronic disease, independent of exercise. Therefore, in this study, we set out to uncover the influence of intrinsic (untrained) aerobic exercise capacity on insulin-stimulated vasoreactivity, a marker of vascular and metabolic health. Specifically, we tested the hypothesis that inherited low-aerobic capacity in LCR rats is associated with vascular dysfunction and impaired vasorelaxation to insulin in the aorta.

## Methods

### Animals

Koch and Britton (Hussain et al. 2001; Koch and Britton 2001; Wisloff et al. 2005) originally developed two lines of rats divergent in intrinsic aerobic endurance exercise capacity. The founder population (N:NIH stock) and generations of the HCR and LCR rats have been previously reported (Wisloff et al. 2005). Briefly, the 13 lowest and 13 highest running capacity rats of each sex were selected from the founder population and randomly paired for mating. By generation six, there was a 171% divergence in running capacity, with most of the change in running capacity relative to the founder population occurring in the HCR line (13% in LCR and 136% in HCR) (Martin et al. 2012). After 28 generations of selection, the HCR and LCR lines differed about eightfold for the running capacity phenotype and maintained considerable narrow-sense heritability (*h*^2^) for this trait (HCR *h*^2^ = 0.47, and LCR *h*^2^ = 0.43) (Ren et al. 2013). Genome-wide 10 K single-nucleotide polymorphism (SNP) genotype data for generations 5, 14, and 26 demonstrated substantial genomic evolution as assessed by Multidimensional Scaling (MDS) analysis. That is, between-line differentiation increased progressively, while within-line diversity decreased. In the present investigation, we used female LCR (*n* = 21) and HCR (*n* = 17) rats from generations 31 and 32, as body weight differences between phenotypes are less marked in females than in males (Hussain et al. 2001), therefore, body weight was less likely to confound our results. At 11 weeks of age, rats were phenotyped for intrinsic endurance running capacity (intrinsic aerobic capacity) by distance run to exhaustion on a motorized treadmill using a velocity-ramped running protocol at the University of Michigan (Hussain et al. 2001; Koch and Britton 2001, 2005). Rats performed three treadmill exercise trials, and the single best trial of three was used as the indicator of aerobic capacity determined by intrinsic genetic composition (Koch and Britton 2005). Following this exercise test, all animals remained sedentary and underwent no further exercise training or testing (spontaneous cage physical activity-only) to factor out the confounding influence of daily exercise. At 16 weeks of age, the animals were shipped to the University of Missouri, where they remained for the completion of the study. Animals were housed in pairs maintained in a temperature (21°C) and light controlled, 12:12-hr light–dark cycle (lights off at 1800 h) animal quarters, and provided *ad libitum* access to water and standard rodent chow (Formulab 5008, Purina Mills, St. Louis, MO) comprised of ∼26% protein, 18% fat, and 56% carbohydrate. The University of Missouri Institutional Animal Care and Use Committee approved all experimental protocols.

### Intraperitoneal Glucose Tolerance Tests

Intraperitoneal glucose tolerance tests (IPGTT) were performed at 39 weeks of age (*n* = 6 per group), as previously described (Rector et al. 2011). Briefly, food was removed from the cages 12 h before each rat received an intraperitoneal injection of dextrose (50% solution, 2 g/kg body weight). Venipuncture blood samples were collected from the lateral tail vein immediately before (0 min) dextrose administration and 15, 30, 45, 60, and 120 min after injection. Insulin and glucose responses were quantified by the product of the area under the curve (AUC) for glucose and insulin using the trapezoidal method (Tai 1994).

### Body Composition and Blood Parameters

Following an overnight fast, we anesthetized the rats at 44 weeks of age with an intraperitoneal injection of sodium pentobarbital (50 mg/kg). We measured body mass of the rats and determined body composition by using a dual energy X-ray absorptiometry machine (Hologic QDR-1000) calibrated for rodents. Subsequently, we harvested tissues, collected blood samples, and euthanized the rats by exsanguination, in full compliance with the American Veterinary Medical Association Guidelines on Euthanasia. Serum samples were prepared by centrifugation and stored at −80°C until analysis. Glucose, cholesterol, triglycerides, and nonesterified fatty acids (NEFA) assays were performed by a clinical diagnostic service provided by the University of Missouri (Comparative Clinical Pathology Services LLC, Columbia, MO) on an Olympus AU680 automated chemistry analyzer (Beckman-Coulter, Brea, CA) using commercially available assays according to the manufacturer’s guidelines. Plasma insulin concentrations were determined using a commercially available, rat-specific ELISA (Alpco Diagnostics, Salem, NH). Samples were run in duplicate and manufacturer’s controls and calibrators were used according to assay instructions. The homeostasis model assessment-insulin resistance (HOMA-IR) was calculated according to the formula of Matthews et al. (Matthews et al. 1985): [fasting glucose (mg/dL) • fasting insulin (*μ*IU/mL)]/405).

### Assessment of Aortic Vascular Function

Isolation and assessment of thoracic aortic ring function was determined as previously described (Thompson et al. 2004; Ingram et al. 2007; Bunker et al. 2010). Briefly, immediately following exsanguination we removed the thoracic aorta and dissected and cleaned the vessel of connective and adipose tissue in ice-cold Krebs-bicarbonate buffer (4°C). The thoracic aorta was segmented into aortic rings, and their outer diameter, inner diameter, and axial length were measured with an Olympus microscope and NIH ImageJ software. Aortic rings were mounted on myographs and submerged in 20-mL water baths containing physiological Krebs solution maintained at 37°C for 1 h to allow for equilibration. We determined the optimal vessel diameter, which elicited a maximal response to 60 mM KCL (L_max_); which was around 150% of the passive diameter. Aortic vasoreactivity was assessed with cumulative concentration-response curves to Acetylcholine (ACh, 10^−10^ to 10^−4^ M), insulin (10–1000 *μ*IU/mL), and sodium nitroprusside (SNP, 10^−10^ to 10^−4^ M). Mounted rings were preconstricted with a submaximal concentration of phenylephrine (PE, 3E^−7^ M) prior to ACh, insulin and SNP dose-response curves.

The vasoreactivity to insulin was assessed with human insulin (Novolin, Novo Nordisk, Plainsboro, NJ) in 10-min intervals. We evaluated the contribution of endothelin-1 (ET-1) and nitric oxide (NO) in the aortic vasomotor response to ACh and insulin by incubating separate aortic rings with tezosentan (3 *μ*M), a nonselective ET_A_ and ET_B_ receptor antagonist, or L-NG-Nitroarginine methyl ester (L-NAME, 300 *μ*M), a NO synthase (NOS) inhibitor, for 20 min prior to ACh and insulin curves. Following each drug dose-response curve, we washed the vessel baths with warm Krebs-bicarbonate buffer (37°C) and allowed a re-equilibration period of 30 min. For ACh, insulin, and SNP curves, relaxation at each concentration was measured and expressed as percent maximum relaxation, where 100% relaxation is equivalent to loss of all tension developed in response to phenylephrine (Bunker et al. 2010).

### RNA extraction and real-time PCR

We performed RNA extraction and real-time PCR on thoracic aorta samples as previously described (Padilla et al. 2013a,b,c; Crissey et al. 2014). Briefly, the thoracic aortic was dissected and all connective and perivascular adipose tissues were removed. The aortic samples were homogenized in TRIzol solution using a tissue homogenizer (TissueLyser LT, Qiagen, Valencia, CA). Total RNA was isolated using the Qiagen’s RNeasy tissue protocol and assayed using a Nanodrop spectrophotometer (Thermo Scientific, Wilmington, DE) to assess purity and concentration. First-strand cDNA was synthesized from total RNA using the High Capacity cDNA Reverse Transcription kit (Applied Biosystems, Carlsbad, CA). Quantitative real-time PCR was performed as previously described (Padilla et al. 2013a,b,c) using the CFX Connect™ Real-Time PCR Detection System (BioRad, Hercules, CA). Primer sequences (Table1) were designed using the NCBI Primer Design tool. All primers were purchased from IDT (Coralville, IA). A 20-*μ*L reaction mixture containing 10 *μ*L iTaq UniverSYBR Green SMX (BioRad, Hercules, CA) and the appropriate concentrations of gene-specific primers plus 4 *μ*L of cDNA template were loaded in each well of a 96-well plate. All PCR reactions were performed in duplicate. PCR was performed with thermal conditions as follows: 95°C for 10 min, followed by 40 cycles of 95°C for 15 s and 60°C for 45 s. A dissociation melt curve analysis was performed to verify the specificity of the PCR products. 18S primers were used to amplify the endogenous control product. Our group has established that 18S is a suitable house-keeping gene for real-time PCR when examining vascular gene expression (Crissey et al. 2014) In this study, 18S CTs were not different among lines. Values of mRNA expression of mRNA are presented as 2^ΔCT^ whereby ΔCT = 18S CT − gene of interest CT (Padilla et al. 2013a,b,c), and normalized to the HCR group of rats, which was set at 1.0.

**Table 1:** Forward and reverse primer sequences for quantitative real-time PCR

Gene	Primer sequence (5 3′)	
	Forward	Reverse
18S	GCCGCTAGAGGTGAAATTCTTG	CATTCTTGGCAAATGCTTTCG
VCAM-1	GAAGGAAACTGGAGAAGACAATCC	TGTACAAGTGGTCCACTTATTTCAATT
ICAM-1	CACAAGGGCTGTCACTGTTCA	CCCTAGTCGGAAGATCGAAAGTC
MCP-1	CTGTCTCAGCCAGATGCAGTTAA	AGCCGACTCATTGGGATCAT
IL-6	AGAGACTTCCAGCCAGTTGC	AGCCTCCGACTTGTGAAGTG
IL-10	CTGGCTCAGCACTGCTATGT	GCAGTTATTGTCACCCCGGA
TNF-*α*	AACACACGAGACGCTGAAGT	TCCAGTGAGTTCCGAAAGCC
ET-1	TTGCTCCTGCTCCTCCTTGAT	TAGACCTAGAAGGGCTTCCTAGT
ENOS	AGGCATCACCAGGAAGAAGA	GGCCAGTCTCAGAGCCATAC
CD8	CACTAGGCTCCAGGTTTCCG	CGCAGCACTTCGCATGTTAG
CD3e	AGTAATGAGCCAGCCGTGTC	ATGCTCCAGAAAGCGTTCCA
CD11c	CTGTCATCAGCAGCCACGA	ACTGTCCACACCGTTTCTCC
CD4	ACCCTAAGGTCTCTGACCCC	TAGGCTGTGCGTGGAGAAAG
F4/80	GCCATAGCCACCTTCCTGTT	ATAGCGCAAGCTGTCTGGTT
PAI-1	AGCTGGGCATGACTGACATCT	GCTGCTCTTGGTCGGAAAGA
FoxP3	CTCCAGTACAGCCGGACAC	GGTTGGGCATCAGGTTCTTG

### Immunoblots

The abdominal aorta was dissected and cleaned of all connective and adipose tissue in ice-cold Krebs-bicarbonate buffer, cut into segments, placed in Laemmli buffer (62.5 mM Tris, pH 6.8, 6 M urea, 160 mM 1,4-dithiothreitol, 2% SDS, and 0.0001% bromophenol blue), flash frozen in liquid nitrogen, and stored at −80°C until analysis. Western blotting to determine protein expression in the abdominal aorta was performed as previously described (Bunker et al. 2010; Mikus et al. 2010). Briefly, samples were subjected to three cycles of boil-vortex-centrifugation and sonication, and protein of was quantified with NanoOrange Protein Quantitation kits (Molecular Probes, Life Technologies, Grand Island, NY). Samples were diluted in Laemmli buffer, boiled for 5 min and equal amounts of aortic proteins (10 *μ*g) were loaded on NuPAGE® Novex® 4–12% Bis-Tris Protein Gels, 1.5 mm, 15 well gels (Life Technologies) under reducing conditions. Proteins were transferred to polyvinyldifluoride membrane (PVDF Hybond-ECL, Amersham). Each gel contained one lane of SeeBlue Plus 2 prestained protein standard (Life Technologies) to evaluate protein transfer and determine molecular weight of the samples. We blocked the membrane for 1 hr at room temperature with 5% nonfat milk in TBS-Tween (20 mM Tris · HCl, 137 mM NaCl, and 0.1% Tween 20), and incubated overnight at room temperature with a primary antibody against: Insulin Receptor-*β* (IR*β*, 1:200, Santa Cruz Biotechnology, Dallas, TX); Endothelin-1 (ET-1, 1:200, Santa Cruz Biotechnology); Endothelin Receptor-A (ET_A_, 1:200, Sigma, St. Louis, MO); Endothelin Receptor-B (ET_B_, 1:250, Alamone Labs); eNOS (1:1000, Transduction Labs); and phospho-specific eNOS (1:200, Transduction Labs). Next, we incubated the membranes with a corresponding horseradish peroxidase-conjugated secondary antibody in a 5% nonfat milk-TBST solution. Protein was detected by enhanced chemiluminescence (Luminata Forte Western HRP Substrate, Millipore) and imaged using a Kodak Image Station 4000R. Total and phospho-specific densities were normalized to HCR values, and the ratio of phospho-eNOS to total eNOS was calculated.

### Drugs and Solutions

All drugs and solutions were obtained from Sigma (St. Louis, MO), except albumin (USB Corporation, Cleveland, OH). The Krebs-bicarbonate buffer solution contained (in mM) 131.5 NaCl, 5.0 KCl, 1.2 NaH2PO4, 1.2 MgCl2, 2.5 CaCl2, 11.2 glucose, 20.8 NaHCO3, 0.003 propranolol, and 0.025 EDTA. The solution was aerated with a 95% O^2^ – 5% CO^2^ mixture (pH7.4) and maintained at 37°C. All drug solutions were prepared in Krebs-bicarbonate buffer, except insulin, which was prepared fresh daily in physiological saline solution with bovine serum album (BSA, 1 g/100 mL). We also prepared SNP fresh daily and protected the serial dilutions from light.

### Data Analysis and Statistics

Results are presented as mean ± SEM. We evaluated overall group effects (LCR vs. HCR) on all dependent variables using two-tailed Student’s *t*-tests. We analyzed all drug dose-response curves from vasomotor function experiments by repeated measures two-way (group × dose) anovas, and evaluated main effects of group, interactions between group and dose, and simple effects. When appropriate, Fisher’s least significant differences (LSD) post hoc analysis was utilized. We used IBM spss Statistics 19 for Windows (Chicago, IL) for all statistical analyses, and established a priori statistical significance of *P *<* *0.05.

## Results

### Animal Characteristics and Glucose Tolerance

**Figure 1:**
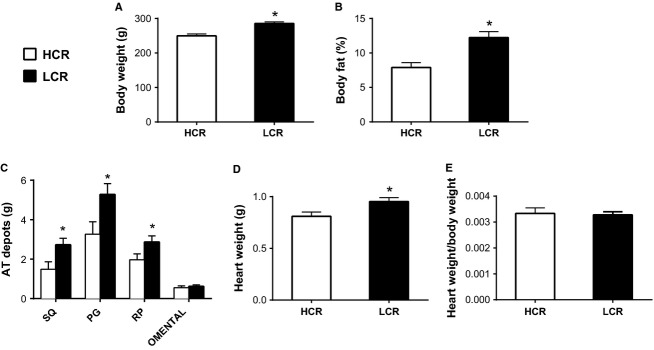
Body composition in low-capacity running (LCR), and high-capacity running (HCR) rats. AT, adipose tissue; SQ, subcutaneous inguinal; PG, perigonadal; and RP, retroperitoneal. Values are expressed as means ± SEM. Body fat, heart weights, and fat pad weights were obtained at 44 weeks of age. *Denotes line difference (*P* < 0.05).

**Figure 2:**
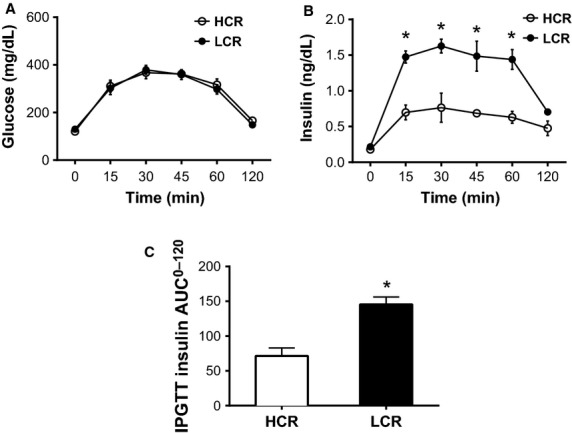
Glucose and insulin responses to an intraperitoneal glucose tolerance test in low-capacity running (LCR), and high-capacity running (HCR) rats, *n* = 6/group at 39 weeks of age. AUC; area under the curve. All data are expressed as means ± SEM. *Denotes line difference (*P* < 0.05).

After 32 generations of artificial selection for intrinsic aerobic capacity, HCR rats ran significantly farther distance (**+**10-fold, *P* **<** 0.001), duration (+5-fold *P* **<** 0.001), and at greater speed (+3-fold *P* **<** 0.001) compared to LCR rats (Table2) during the graded exercise tests to exhaustion, performed at 11 weeks of age. For the remainder of the study, the rats did not engage in any structured exercise training or exercise bouts. LCR rats were ∼13% heavier, owing to ∼35% greater percent body fat compared to HCR animals determined by DEXA (Fig.1A and B). The increased adiposity in the LCR rats was evident by ∼34% more visceral (perigonadal and retroperitoneal) adipose tissue than HCR rats (Fig.1C). Although heart weight was slightly different between lines (Fig.1D), no differences were observed when heart weight was normalized to body weight (Fig.1E). Fasting serum triglycerides, nonesterified fatty acids, glucose, insulin, and HOMA-IR were all not significantly different between LCR and HCR female rats at 44 weeks of age (Table2, *P* > 0.05). Glucose responses during the IPGTT were also not different between lines (Fig.2A and D); however, insulin secretion (Fig.2B) and insulin AUC (Fig.2C) were greater in LCR animals suggestive of modestly impaired insulin sensitivity.

**Table 2:** Animal characteristics

Variable	LCR	HCR
Max distance run to exhaustion at 11 weeks (m)	238 ± 8	2303 ± 68*
Time to exhaustion at 11 weeks (min)	17.2 ± 0.4	78.7 ± 1.4*
Speed at exhaustion at 11 weeks (m/min)	18.1 ± 0.2	48.8 ± 0.7*
Triglycerides (mg dL^−1^)	44.9 ± 6.8	29.2 ± 2.7
Nonesterified fatty acids (mmol L^−1^)	44.3 ± 3.3	39.9 ± 2.0
Glucose (mg dL^−1^)	150 ± 5	173 ± 15
Insulin (ng dL^−1^)	1.74 ± 0.29	1.78 ± 0.46
HOMA-IR index	0.67 ± 0.11	0.87 ± 0.33

Values are expressed as means ± SEM.

* Denotes line difference (*P* < 0.05).

LCR, low-capacity runner; HCR, high-capacity running; HOMA-IR, homeostasis model assessment of insulin resistance.

### Aortic Gene Expression

**Figure 3:**
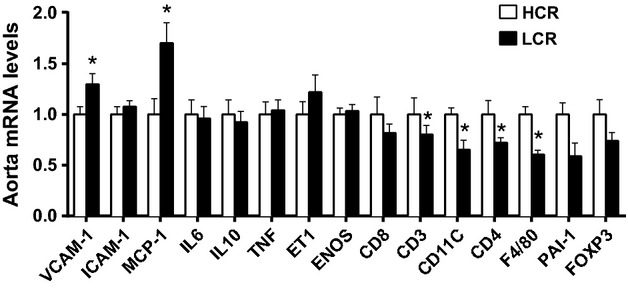
Aortic gene expression profile of several inflammatory and adhesion molecule-related genes in low-capacity running (LCR), and high-capacity running (HCR) rats. Values are expressed as means ± SEM. For each gene, HCR is used as the reference group and set at 1. *Denotes line difference (*P* < 0.05).

As shown in Fig.3, LCR rats exhibited increased mRNA expression of the chemoattractant molecules VCAM-1 and MCP-1 but reduced expression of inflammatory genes CD11C, CD4, F4/80, and PAI-1 relative to HCR (Fig.3, all *P* < 0.05). The up-regulation of pro-atherogenic/inflammatory chemoattractant genes may be suggestive of an increased atherosclerosis risk within the aortic wall of LCR.

### Aortic Protein Expression

**Figure 4:**
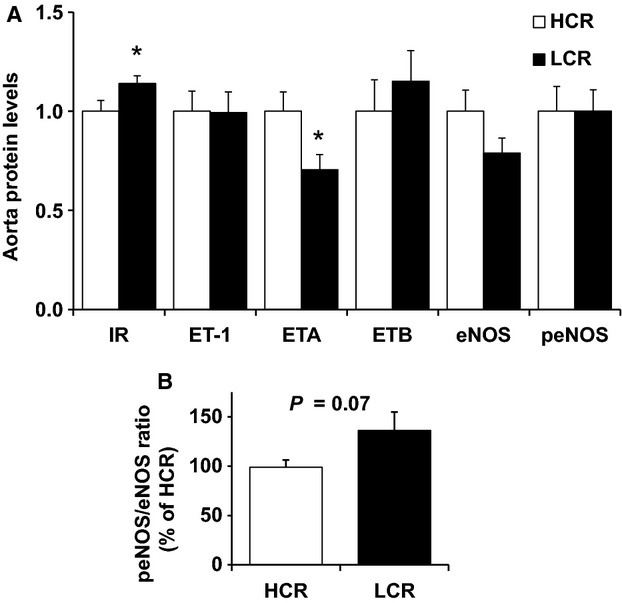
Aortic protein expression of several vascular insulin signaling peptides in low-capacity running (LCR), and high-capacity running (HCR) rats. Values are expressed as means ± SEM. For each protein, HCR is used as the reference group and set at 1. *N* = 14/group *Denotes line difference (*P* < 0.05).

Immunoblot analysis presented in Figure4A revealed greater Insulin Receptor (IR) and lower Endothelin Receptor-A (ET_A_) protein expression in the abdominal aorta of LCR compared to HCR animals (*P* < 0.05). However, we observed no differences in Endothelin-1 (ET-1) or Endothelin Receptor-B (ET_B_) protein expression between lines. Insulin induces vasocontraction by ET-1 via either ET_A_ or ET_B_ receptors on vascular smooth muscle, while activation of ET_B_ receptors on endothelium signals vasorelaxation through release of NO by the endothelium (Kim et al. 2006). Thus, our results suggest that a greater relative density of endothelial ET_B_ receptors in LCR, may have promoted greater vasorelaxation through production of NO. Examination of the p-eNOS/eNOS ratios (Fig.4B) showed that, relative to the total pool of available eNOS in the abdominal aorta, the percentage of p-eNOS trended (*P* = 0.07) to be greater in LCR rats compared with HCR rats.

### Aortic Vasoreactivity

**Figure 5:**
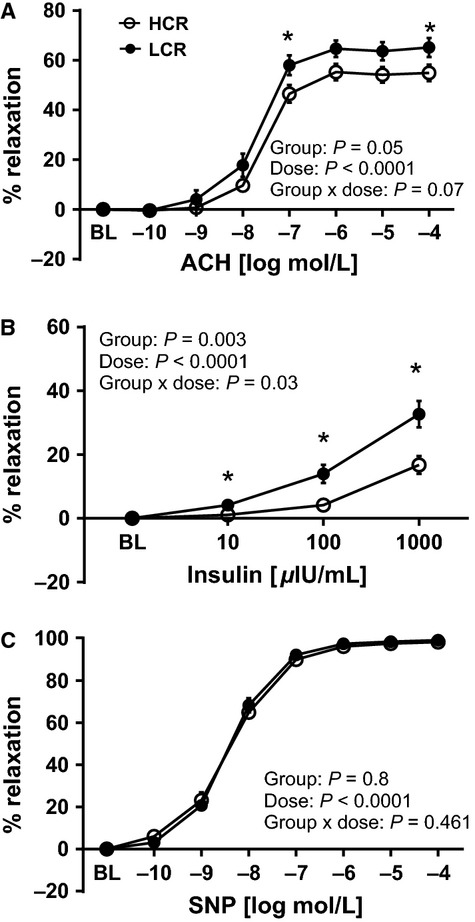
Vasomotor function of thoracic aortic rings in low-capacity running (LCR), and high-capacity running (HCR) rats. Values are expressed as means ± SEM. *Denotes line difference (*P* < 0.05).

We observed no differences in vessel characteristics: outer diameter, inner diameter, vessel length, or specific tension (normalized to vessel surface area) elicited by 60 mM KCl, and phenylephrine preconstriction between lines (Table3). Counter to our hypothesis endothelium dependent dilation, assessed by acetylcholine (ACh)-induced vasorelaxation in thoracic aortic rings was significantly greater in LCR compared to HCR animals (Fig.5A, Group effect *P* < 0.05). The maximum aortic relaxation to ACh was 55.0 ± 3.3% in HCR, and 65.2 ± 3.8% in LCR rats. Similarly, insulin-stimulated aortic vasorelaxation was significantly greater in LCR than HCR across all insulin doses (Fig.5B, Group *P* = 0.003) with a significant group × dose interaction (*P* = 0.03). Insulin (1000 *μ*IU/mL) induced approximately twofold higher maximal vasorelaxation of the aorta in LCR (32.7 ± 4.1%) compared to HCR (16.7 ± 2.8%). We did not observe any differences in aortic vascular smooth muscle function, as assessed by SNP-mediated relaxation (Fig.5C).

**Table 3:** Vessel characteristics

Group	OD (mm)	ID (mm)	Length (mm)	SA (mm^2^)	Wall Thickness (mm)	60 mM KCL (g)	Pre-PE (g)
LCR	1.74 ± 0.02	1.34 ± 0.02	2.43 ± 0.04	0.97 ± 0.03	0.20 ± 0.01	5.0 ± 0.2	4.1 ± 0.1
HCR	1.75 ± 0.02	1.39 ± 0.02	2.50 ± 0.05	0.90 ± 0.03	0.18 ± 0.01	5.2 ± 0.2	4.2 ± 0.1

Values are expressed as means ± SEM.

LCR, low-capacity runner; HCR, high-capacity runner; OD, outer diameter of the aorta; ID, inner diameter of the aorta; SA, aorta surface area; 60 mM KCL, contraction tension in response to 60 mM potassium chloride; and Pre-PE, preconstriction tension produced by phenylephrine.

### Role of Endothelin-1 and Nitric Oxide in Aortic Vasomotor Responses

**Figure 6:**
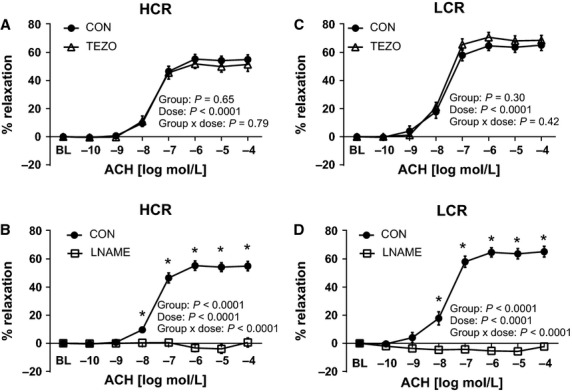
Effects of endothelin-1 receptor blockade with tezosentan (TEZO) and nitric oxide synthase inhibition (L-NAME) on acethylcholine (ACh)-induced vasorelaxation of thoracic aortic rings of low-capacity running (LCR) and high-capacity running (HCR) rats. The left panel (A and B) present data from ACh dose-response curves in HCR, and right panel (C and D) present data from ACh dose-response curves in LCR. Closed circles represent % relaxation in response to ACh alone, open triangles represent % relaxation to ACh in the presence of 3 *μ*M tezosentan, a nonspecific inhibitor of ET-1 receptors (top panel, A and C) *N* = 9–16/group, and open squares represent % relaxation to insulin in the presence of 300 *μ*M L-NAME, a nitric oxide synthase inhibitor, (bottom panel, B and D) *N* = 6–8/group. Values are expressed as means ± SEM *Denotes line difference (*P* < 0.05).

**Figure 7:**
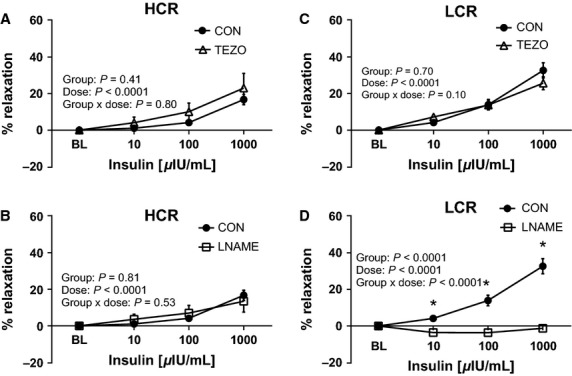
Effects of endothelin-1 receptor blockade with tezosentan (TEZO) and nitric oxide synthase inhibition (L-NAME) on insulin-mediated vasorelaxtion of thoracic aortic rings of low-capacity running (LCR) and high-capacity running (HCR) rats. The left panels (A and B) present data from insulin dose-response curves in HCR, and right panel (C and D) present data from insulin dose-response curves in LCR. Closed circles represent % relaxation in response to insulin alone, open triangles represent % relaxation to insulin in the presence of 3 *μ*M TEZO a nonspecific inhibitor of ET-1 receptors (top panel, A and C) *N* = 9–16/group, and open squares represent % relaxation to insulin in the presence of 300 *μ*M L-NAME, a nitric oxide synthase inhibitor, (bottom panel, B and D) *N* = 6–8/group. Insulin *μ*IU/mL, micro-international units per milliliter. Values are expressed as means ± SEM. *Denotes line difference (*P* < 0.05).

ACh-induced aortic vasorelaxation was unaltered by ET-1 blockade, with tezosentan (Fig.6A and C) in both lines. In contrast, inhibition of NOS with L-NAME completely blocked aortic ring relaxation responses to ACh in both LCR and HCR animals, indicating that ACh-mediated vasorelaxation in the thoracic aorta is entirely NO mediated (Fig.6B and D), corresponding to previously reported data in this animal model (Ritchie et al. 2013). Analogous to ACh, ET-1 blockade (3 *μ*M tezosentan) had no effect on insulin-stimulated relaxation in the aorta of LCR or HCR animals (Fig.7A and C), suggesting that insulin-induced ET-1 release and/or ET-1-mediated contraction does not account for differences in the vasomotor response to insulin between lines. In contrast, inhibition of NOS with L-NAME completely abolished insulin-mediated vasorelaxation of the aorta in LCR animals (Fig.7D), with no effect in HCR rats (Fig.7B). Importantly, these data suggest that insulin-induced aortic relaxation of HCR rats is not driven by mechanisms involving eNOS generated NO, while insulin-stimulated vasorelaxation of the aorta is entirely NO dependent in LCR rats.

## Discussion

Evidence in humans indicates that low-aerobic capacity is a strong predictor of early mortality (Blair et al. 1989a, 1989b; Myers et al. 2002) and is linked to cardiovascular disease (Myers et al. 2002), yet the mechanism(s) are not completely understood. In this study, we investigated the relationship between intrinsic aerobic exercise capacity and vascular insulin reactivity in a rodent model divergent in endurance running capacity (LCR and HCR rats) known to display differing metabolic profiles (Noland et al. 2007; Lessard et al. 2009; Naples et al. 2010) by testing the hypothesis that inherited low-aerobic capacity is associated with vascular dysfunction and impaired vasorelaxation. Our main findings include: (1) aortic endothelial dependent vasorelaxation, assessed by ACh, was similar if not greater in LCR compared to HCR rats; (2) insulin-stimulated vasorelaxation of the aorta was greater in LCR compared to HCR rats; (3) the contribution of NO in mediating insulin-stimulated vasorelaxation was greater in LCR compared to HCR rats; (4) the aorta of LCR rats displayed greater expression of IR protein and lower vasoconstrictive ET_A_ receptor protein; and (5) the aorta of LCR rats exhibited increased mRNA expression of VCAM-1 and MCP-1 but decreased mRNA for markers of immune cell infiltration (i.e., decreased CD3, CD4, CD11C, F4/80).

We chose to assess the influence of intrinsic aerobic capacity on endothelial function, because it has been shown to be an independent predictor of long-term cardiovascular disease progression and cardiovascular events (Schachinger et al. 2000). To assess endothelial function we examined acetylcholine (ACh)-induced vasorelaxation in thoracic aortic ring segments isolated from LCR and HCR rats. Counter to our hypothesis, and previous reports of endothelial dysfunction in the carotid artery of female LCR rats (Wisloff et al. 2005), our results indicate that maximal ACh-induced vasorelaxation in the aorta was similar and even slightly greater (10%) in LCR compared to HCR animals. Analogous to our findings, Ritchie et al. reported no differences in aortic ACh-induced vasorelaxation in HCR or LCR rats (Ritchie et al. 2013). Moreover, the female LCR and HCR rats in the studies of Wisloff et al., Ritchie et al., and our study varied in age (ranging from 16 to 24 weeks (Wisloff et al. 2005); 35 weeks (Ritchie et al. 2013); and 44 weeks in our study), a known modifier of endothelial function. Taken together, these data suggest that the carotid artery may be more susceptible to endothelial dysfunction than the aorta in LCR rats, and that the age-related decline in endothelial function appears to differ between lines. To our knowledge, no comprehensive time course study has been conducted to determine the effects of age on endothelial function in these two lines of rats. Our results show that aortic endothelial dependent NO-mediated vasorelaxation is similar, if not greater in LCR than HCR rats.

Contrary to our primary hypothesis, low-aerobic capacity was not associated with impaired insulin-stimulated vasorelaxation of the aorta in LCR rats; we observed greater aortic insulin-stimulated vasorelaxation in LCR rats compared to HCR animals. Insulin’s cardiovascular actions are complex and are thought to be balanced by two primary endothelial insulin signaling pathways to induce: (1) vasorelaxation via the endothelial nitric oxide synthase (eNOS) pathway produce nitric oxide (NO) (Clark et al. 2003; Vincent et al. 2003; Kim et al. 2006), and (2) vasocontraction via mitogen-activated protein kinase (MAPK) signaling to produce endothelin-1 (ET-1) (Kim et al. 2006; Muniyappa and Quon 2007; Muniyappa et al. 2007). Thus, we evaluated the roles of NO and ET-1 in insulin-mediated aortic vasorelaxation. Remarkably, inhibition of nitric oxide synthase (NOS) with L-NAME entirely abolished insulin-mediated vasorelaxation in the aorta of LCR animals, with no effect in HCR, suggesting that low-aerobic capacity is associated with greater NO reliance to stimulate insulin-induced aortic vasorelaxation. While insulin has been shown to increase eNOS mRNA and protein expression and downstream NO production in humans and rodents (Aljada and Dandona 2000; Kuboki et al. 2000; Fisslthaler et al. 2003), we did not observe any significant differences in eNOS mRNA or protein levels between LCR and HCR. Despite similar eNOS levels, the ratio of phosphorylated eNOS to total eNOS in the LCR aorta trended to be 37% greater than HCR (Fig.4B), suggesting that enhanced relative phosphorylation (activation) of eNOS in the aorta of LCR rats may have contributed to greater NO production. Insulin-stimulated ET-1 aortic contraction did not differ between lines, suggesting that local vascular ET-1 release and/or ET-1 mediated vasocontraction does not account for the differences in the aortic vasomotor response to insulin in LCR and HCR rats. Insulin-induced ET-1 vasocontraction occurs through either ET_A_ or ET_B_ receptors on vascular smooth muscle, while activation of ET_B_ receptors on endothelium signals vasodilation through release of NO by the endothelium (Kim et al. 2006). While ET-1 and ET_B_ receptor protein expression was not different between lines, ET_A_ receptor expression was lower in the LCR aorta wall. Together, these data suggest that LCR aorta has a greater relative density of endothelial ET_B_ receptors, which may promote vasorelaxation through production of NO. In summary our results suggest that vascular insulin signaling in the LCR aorta may have greater reliance on NO via both the eNOS and ET-1 pathways leading to greater insulin-stimulated vasorelaxation.

Similar to our prior investigations LCR rats displayed elevated insulin secretion during a glucose tolerance test (Noland et al. 2007; Naples et al. 2010). We also found greater insulin receptor (IR) protein expression, and a trend for greater the relative activation of eNOS (p-eNOS/total eNOS ratio) in the LCR aorta. Although insulin has been shown to increase the expression of eNOS mRNA and protein in human and animal vascular cells (Aljada and Dandona 2000; Kuboki et al. 2000; Fisslthaler et al. 2003), we did not observe differences in basal eNOS mRNA or protein levels between LCR and HCR. Thus, our results are consistent with the possibility that insulin-stimulated expression of total or phosphyorylated eNOS may be up-regulated in the LCR aorta. We did not perform insulin-stimulated experiments for immunoblot analysis in this study. Overall our results suggest that increased insulin-stimulated vasorelaxation in the aorta of LCR rats may be the result of enhanced vascular insulin sensitivity, via greater vascular IR, and altered vascular insulin signaling pathways that modestly increased the relative phosphorylation of eNOS and NO production.

Alternatively, it is possible that the higher skeletal muscle capillarity and mitochondrial content found in high-aerobic capacity reduces the vascular reactivity to insulin necessary to maintain metabolic peripheral insulin action. That is, it is possible that the vascular actions of insulin with high-aerobic capacity may be less important for optimal delivery of insulin and glucose, due to the greater absolute skeletal muscle perfusion and muscle insulin sensitivity. In addition, it is possible that cage confinement may induce more behavioral stress in the “bred to run” HCR than LCR rats, which may have influenced vascular function in our study; however, our results do not allow us to evaluate this possibility.

Our finding that NOS inhibition did not affect insulin-stimulated vasorelaxation in HCR rats is surprising given current understanding of insulin’s vascular actions to elicit vasorelaxation via NO (Clark et al. 2003; Vincent et al. 2003; Kim et al. 2006). In addition to the endothelial actions of insulin, insulin has been shown to promote vasorelaxation via direct actions in vascular smooth muscle cells (Sowers 2004). The design of our study does not allow us to determine the relative contribution of the endothelium and smooth muscle in the vasoreactivity to insulin in the aorta, hence we cannot rule out the influence of vascular smooth muscle to our results. Rather, we deduce that the vascular actions of insulin in the aorta are primarily endothelial effects based on prior data, where we showed that that insulin-mediated vasorelaxation is completely abolished in the denuded rat aorta (Padilla and Jenkins 2013). Our data indicate that ACh and insulin-stimulated vasomotor relaxation in the LCR aorta is entirely through eNOS-mediated mechanisms. In accord with our findings, Ritchie et al. report that EDHF-mediated ACh vasorelaxation is impaired in LCR, despite intact NO-mediated relaxation (Ritchie et al. 2013). Taken together, it seems possible that insulin’s ability to stimulate PGI_2_ and EDHF pathways is reduced with low-aerobic capacity, leading to a compensatory overactivation of insulin-stimulated NO production. Unlike LCR, insulin-induced vasomotor relaxation via NO-independent mechanisms in the HCR aorta. Future work investigating mechanisms of vascular insulin action in endothelial and vascular smooth muscle cells in this model are warranted.

To further characterize the vascular phenotype in LCR and HCR rats, we assessed markers of inflammation and immune cell infiltration in the aortic wall. We assessed CD11c, a marker of classically activated macrophages/antigen presenting cells; CD3, a T lymphoycte marker; CD4, a T helper cell marker; F4/80, a global macrophage marker; and vascular adhesion molecules VCAM-1 and MCP-1. Concomitant with a down-regulation of markers of immune cell infiltration (CD11c, CD3, CD4, and F4/80,) we observed an up-regulation of inflammatory genes (VCAM-1 and MCP-1) in the aorta of LCR. The enhanced expression of adhesion molecules along with reduced immune infiltrates may represent an early snapshot of immune activation in the aorta wall, such that a future time point might exhibit enhanced immune cell infiltration in LCR. Overall, the expression pattern of pro-inflammatory markers together with an anti-atherogenic immune cell profile found with low-aerobic capacity requires further investigation.

In summary, we provide evidence that selective breeding for intrinsic aerobic capacity, in the absence of exercise training, modulates vasomotor function and vascular phenotype in the aorta of mature female rats. Counter to our hypothesis, we found that LCR rats exhibit similar, if not greater endothelial dependent NO-mediated vasorelaxation compared to HCR rats, suggesting that reduced endothelial function in the aorta is not always associated with decreased aerobic capacity. Furthermore, low-aerobic capacity was associated with greater NO-mediated aortic insulin-stimulated vasorelaxation, a phenomenon that appears to be compensatory. Remarkably, insulin-mediated vasorelaxation in HCR rats was independent of NO, suggesting that high-aerobic capacity is associated with a redundancy in mechanisms driving insulin-mediated vascular effects. In conclusion, contrary to our hypothesis, we found that low-aerobic capacity was associated with enhanced aortic endothelial function and NO-mediated reactivity to insulin, despite increased adiposity and evidence of whole body insulin resistance.
